# Association of Angiogenic and Inflammatory Markers with Power Doppler Ultrasound Vascularity Grade and DAS28-CRP in Early Rheumatoid Arthritis: A Comparative Analysis

**DOI:** 10.1155/2018/6906374

**Published:** 2018-11-25

**Authors:** Sanchaita Misra, Sumantro Mondal, Sudipta Chatterjee, Aharna Guin, Pradyot Sinhamahapatra, Alakendu Ghosh

**Affiliations:** ^1^M.Sc, Junior Research Fellow, Department of Rheumatology, Institute of Postgraduate Medical Education and Research, SSKM Hospital, Kolkata, India; ^2^DM (Rheumatology), Medical Officer, Department of Rheumatology, Institute of Postgraduate Medical Education and Research, SSKM Hospital, Kolkata, India; ^3^M.Sc, Senior Research Fellow, Department of Rheumatology, Institute of Postgraduate Medical Education and Research, SSKM Hospital, Kolkata, India; ^4^Ph.D., Senior Research Fellow, Department of Rheumatology, Institute of Postgraduate Medical Education and Research, SSKM Hospital, Kolkata, India; ^5^MD (General Medicine), Associate Professor, Department of Rheumatology, Institute of Postgraduate Medical Education and Research, SSKM Hospital, Kolkata, India; ^6^DNB (Medicine), FRCP, Professor and Head, Department of Rheumatology, Institute of Postgraduate Medical Education and Research, SSKM Hospital, Kolkata, India

## Abstract

**Objective:**

Upregulation of various proinflammatory and angiogenic mediators orchestrates the typical pathological synovial alterations in rheumatoid arthritis (RA). DAS28-CRP is commonly used for assessment of RA disease activity and power Doppler ultrasonography (PDUS) is an important modality for assessing synovial vascularity. This study was intended to look for the association of various inflammatory and angiogenic mediators, with respect to different PDUS vascularity grades and disease activity status, in early RA patients.

**Methods:**

50 early RA patients (<6 months disease duration) with either moderate or high disease activity and 30 healthy controls were included in this study. RA patients were subcategorized based on PDUS vascularity grading of wrist joints. Serum levels of proinflammatory cytokines [tumor necrosis factor-*α* (TNF- *α*); interleukin-6(IL-6)] and angiogenic markers [angiopoietin-1 and 2 (Ang-1 and Ang-2); vascular endothelial growth factor (VEGF) ] were measured and compared among different patient subgroups.

**Results:**

Among 50 patients, 22 and 28 patients had moderate and high DAS28-CRP score, respectively. Patients with grade 3 PDUS score, even with moderate DAS value, showed a significant rise in Ang-1 (*p*<0.02), VEGF (*p*<0.008), Ang-2 (*p *<0.001), and TNF-*α* (*p*<0.005) level compared to grade 2 PDUS patients with high DAS values.

**Conclusion:**

Higher serum level of angiogenic and inflammatory markers was noted among patients with moderate disease activity but with advanced PDUS vascularity (grade 3) in comparison to high disease activity group with less severe PDUS vascularity (grade 2). PDUS vascularity grading better reflects some markers of angiogenesis and inflammation, than composite disease activity index.

## 1. Introduction

Rheumatoid arthritis (RA) is a chronic autoimmune disease that manifests as synovitis of multiple joints and may eventually progress to joint destruction. Abnormal synovial proliferation due to cellular recruitment, angiogenesis, and most importantly pannus formation is the pathologic hallmarks of RA. Upregulation of multiple proinflammatory and angiogenic mediators in hypoxic RA synovium initiates and perpetuates synovial inflammatory process in RA [1]

Early diagnosis, evaluation of disease activity, and administration of conventional synthetic or biologic DMARDs may restrict the disease progression. The treatment of RA is mostly guided by assessment of disease activity state. At present, various parameters are used to quantify the inflammatory burden (or the level of disease activity), such as the number of joints affected, ESR, CRP, or a composite of these, like disease activity score (DAS-28) [2, 3]. Some authors proposed that DAS28 ESR and CRP cut-off values are not interchangeable [4]. The cut-off values of DAS28 CRP should be modified and they may be lower than the DAS-28 ESR. However, DAS 28 may not be adequate enough to reflect the underlying inflammatory activity in RA. [5, 6]

Power Doppler ultrasonography (PDUS) has been established as a sensitive modality for evaluating disease activity in RA [7, 8]. PDUS is a reliable tool to detect synovial vascularity in patients with inflammatory arthritis and ultrasonographically assessed synovial proliferation and vascularity correlates with RA disease activity. [9, 10]. Conversely, some other studies showed that PDUS vascularity grading of synovial joints in RA patients might not correlate with DAS 28 derived disease activity status [11]. Some reports pointed out the positive relationship of PDUS synovitis grading with the markers promoting angiogenesis in RA synovium [12].

Vascular endothelial growth factor (VEGF) is an important growth factor favoring synovial angiogenesis and progression of joint inflammation in RA. The crucial role of VEGF in the pathogenesis of early RA patients has been well documented in literatures [13, 14]. An elevated level of VEGF is found in both serum and synovial fluid of RA patient [15, 16]. Angiopoietins are another group of angiogenic mediators that augments the inflammatory process in RA by promoting angiogenesis. Angiopoietin-1 (Ang-1) and Angiopoietin-2 (Ang-2) are over expressed in RA synovium [17]. Synergistically VEGF and Angiopoietins carries important pathogenic role in early RA by stabilizing and remodeling of newly formed synovial blood vessels. [18]

In this context, our study was intended to find the relationship between PDUS vascularity grading with the markers of angiogenesis and inflammation in early RA patients. Furthermore, a comparative analysis was done between PDUS vascularity grading and DAS-28CRP score to study their association with the expression of these markers.

## 2. Materials and Methods

### 2.1. Patients and Controls

This cross-sectional, observation study was conducted form (February 2015-May 2017). Fifty early RA patients were recruited from the Rheumatology clinic at the Institute of Post Graduate Medical Education and Research, SSKM Hospital, Kolkata, India, along with 30 healthy ages and sex matched volunteered controls. Informed consent was taken from all the study participants. The study was approved by Institutional Ethics Committee of the SSKM hospital according to the declaration of Helsinki.

All patients fulfilled the following inclusion criteria:

1. Diagnosed as RA by a rheumatologist as per ACR/EULAR classification criteria for RA 2010. [19]
2. Disease activity moderate and high (DAS-28 CRP).
3. Disease duration of less than 6 months.
4. No prior use of disease modifying antirheumatic drugs and corticosteroids.
5. Clinical evidence of wrist joint involvement.

### 2.2. Baseline Evaluation of RA Disease Activity and Laboratory Tests

Baseline demographic parameters were recorded. In RA group, disease activity was measured by DAS-28CRP and approved cut-off values were used to stratify them moderate and high disease activity states. Modified cut-off points for DAS-28 CRP were 2.3, 2.7, and 4.1 for remission, low disease activity, and high disease activity, respectively [4]. We also analyzed our result based on conventional cut-off values,<2.6 for remission, 2.6 to ≤3.2 for low disease activity, >3.2 to ≤ 5.1 for moderate disease activity, and > 5.1 for high disease activity. HAQ-DI score and visual analog scales for global assessment of pain (0-100 scale) were also recorded. Clinical evaluations were performed by a rheumatologist prior to laboratory testing of inflammatory and angiogenic markers and ultrasound examination. Rheumatoid factor (RF) and high sensitivity C reactive protein (hs-CRP) were measured by Nephelometry. Anti-citrullinated peptide antibody (ACPA) level was measured by ELISA method.

### 2.3. Assessment of Wrist Joint for Synovial Vascularity

**Figure 1:**
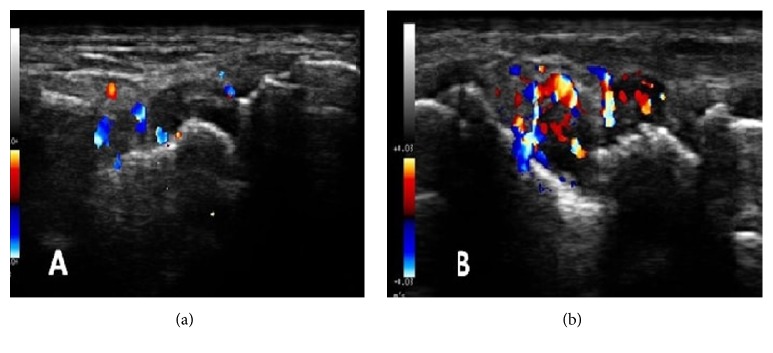
Representative PDUS images of different grades of synovial vascularity of wrist joint ((a): Grade II and (b): Grade III vascularity).

PDUS of wrist joints were performed by a radiologist experienced in musculoskeletal ultrasound, blinded to clinical and laboratory details of the patients. My Lab 25 gold, E-saote USG platform was used. The assessments were performed using a high frequency linear array transducer (frequency range 12 to 18 MHz). Doppler signal gain, filter, and pulse repetition frequency (PRF) were set optimally for each patient. In each patient, the study was performed at dorsal aspect of bilateral wrist joints in 2 planes (longitudinal and transverse). PDUS vascularity grading was done as per OMERACT definition. Synovial vascularity by PDUS was graded as follows: grade 0 = no flow signal in the synovium, grade 1= up to 3 single spots signals or up to 2 confluent spots or 1 confluent spot + up to 2 single spots, grade 2= vessel signals in less than half of the area of the synovium(<50%), and grade 3= vessel signals in more than half of the area of the synovium (>50%) [20]. PDUS grade 2 and grade 3 synovial vascularity has been demonstrated in Figure 1.

### 2.4. Detection and Quantification of Inflammatory and Angiogenic Markers

Ang-1 (Raybiotech, USA), Ang-2 (Raybiotech, USA), and VEGF-A (Raybiotech, USA) from serum were analyzed by ELISA following the kit protocols. TNF-*α* (Raybiotech, USA) and IL-6 (Raybiotech, USA) were similarly analyzed following the suppliers' protocols.

### 2.5. Statistical Analysis

All normally distributed data were tested by applying Shapiro-Wilk test. Normally distributed variables were presented as mean value with standard deviation (SD). DAS-28CRP was found to be nonnormally distributed and was presented as median (interquartile range). The difference between groups was estimated by independent *t* test and Man-Whitney *U* test for normally distributed and skewed data, respectively. *χ*^2^ test was used for comparing categorical variables. Logistic regression analysis was done to predict relationship between PDUS and angiogenic markers and multiple linear regression analysis was done to predict relationship between angiogenic markers and DAS-28CRP. All *p* values <0.05 were considered as statistically significant. All statistical analyses were performed using (GraphPad prism v 5.0 and MedcalC v 11.6).

## 3. Results

### 3.1. Baseline Characteristics and Laboratory Parameters of Study Population

Mean age of the patients was 35 years. Majority of them were female (46/50). There was no significant difference in terms of mean age, gender ratio, and BMI between RA and control group. Clinical and serological parameters of the studied patients have been demonstrated in Table 1.

### 3.2. PDUS Analysis and Gradation of Vascularity

Based on conventional DAS-28 scores 24 patients were categorized as moderate disease activity and 26 patients with high disease activity. According to the modified cut-off value of DAS-28CRP, 2 patients from moderate disease activity were recategorized as high disease activity status. Among 24 patients with moderate DAS, 11 patients were categorized under grade 2 PDUS vascularity and 13 patients were categorized under grade 3 PDUS vascularity. In high DAS group, 12 patients were categorized under grade 2 PDUS vascularity and 14 patients were categorized under grade 3 PDUS vascularity. According to modified DAS-28CRP cut-off value, 10 of the 22 patients with moderate DAS showed grade 2 PDUS vascularity and 12 patients showed grade 3 PDUS vascularity. Among 28 patients with high DAS score, grade 2 and grade 3 PDUS vascularity were noted in 13 and 15 patients, respectively. There was slight difference in number of patients with moderate and high disease activity between conventional DAS-28CRP and the modified cut-off value of DAS-28CRP.

### 3.3. Expression of Inflammatory and Angiogenic Markers in Both High and Moderate DAS Patients

**Figure 2:**
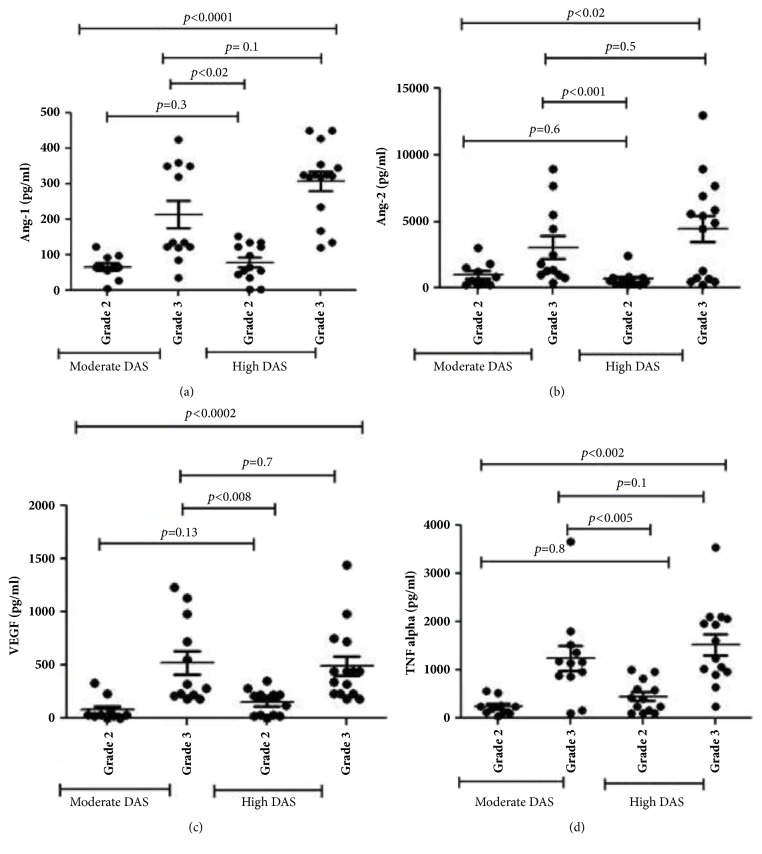
Scatter plot illustration histograms showing levels of different angiogenic and inflammatory mediators within various study subgroups. (a) Ang-1, (b) Ang-2, (c) VEGF, and (d) TNF-*α* (values are mean±SD).

Angiogenic and inflammatory markers were high in RA patients in comparison to control group (Table 2). Patients with moderate DAS values and grade 3 PDUS vascularity had a significant elevated levels of ANG- 1(*p*<0.02), ANG-2(*p*<0.001), and VEGF (*p*<0.008) compared to patients with high DAS, grade 2 PDUS vascularity (Figure 2). When reanalysis was done based on the conventional cut-off values of DAS-28CRP for the same parameters, statistical significance was maintained (Ang1 (*p*<0.02), ANG-2 (*p*<0.001), and VEGF (*p*<0.005) (Figure not shown)).

Among the inflammatory markers, TNF-*α* level was significantly elevated in all RA patients. However, patients with grade 3 PDUS vascularity among moderate DAS had significantly increased TNF-*α* level than those with grade 2 PDUS vascularity among high DAS (*p*<0.005). Statistical significance also maintained when conventional cut-off values of DAS-28CRP were applied (*p*.<0.03). No significant difference in IL-6 level (*p*=0.1) could be observed between the above-mentioned PDUS groups of moderate and high DAS patients.

### 3.4. Logistic Regression Analysis Models to Compare PDUS Vascularity Grade with Angiogenic and Inflammatory Markers in Early RA

Binary logistic regression (LR) analysis was performed to assess which of the biomarkers influenced the risk of showing PDUS grade. The variables included in the LR analysis were Ang1, Ang2, TNF–*α*, and VEGF. All variables were numerical in nature and were entered simultaneously.

Overall model quality was fair (r^2^ =0.5320) (Table 3(a)) which indicate a moderate predictive accuracy.

The power of the model's predicted values to discriminate between positive and negative cases has been quantified by the area under curve value which, at 0.846, is close to 1 and thus indicates high discriminating power. The cases that convert to PDUS have been correctly predicted to the extent of 78.72%.

The LR analysis indicates that VEGF and TNF-*α* are the factors predicting severity of PDUS.

### 3.5. Multiple Linear Regression Models to Compare DAS 28-CRP with Angiogenic and Inflammatory Markers in Early RA

Multiple regression analysis between various angiogenic and inflammatory markers did not reflect any significant association of DAS-28CRP (r^2^ 0.02) [Table 3(b)].

## 4. Discussion

Angiogenesis is an integral component of synovial joint inflammation. It starts at a very initial stage of RA, under the influence of various inflammatory cytokines, chemokines, and growth factors [21]. It exhibits a pivotal role in perpetuating joint inflammation. Early RA patients were classified into moderate and high disease activity groups based on DAS-28 CRP score. These two groups were included in our study as these activity states are associated with unfavorable long term outcomes in RA patients than the low DAS 28 state at baseline. Subcategorization of study population based on PDUS vascularity grades and assessment of angiogenic, inflammatory markers within these groups was done to determine the adequacy of DAS-28CRP grading in evaluating the underlying inflammatory activity in early RA. We found a subgroup of early RA patients with moderate DAS-28 CRP score, in whom the angiogenic and inflammatory mediators were actually over expressed than patients with high DAS-28 CRP score. As some authors proposed that DAS-28 ESR and CRP cut-off values may not be similar and they may be lower for DAS-28 CRP, so all the analyses were conducted based on conventional and modified cut-off values for the DAS-28CRP, as suggested by Inoue E. et al. [4]. However there was no significant change in the study result. Overexpression of these markers showed a positive association with PDUS vascularity grading, except for ANG-1 and ANG-2. This finding is to some extent in contradiction to the current knowledge, as these are considered to be inducers of destabilized angiogenesis in inflammatory arthritis. DAS-28CRP did not show any significant association with respective angiogenic and inflammatory markers. Hence, in early RA, DAS28 CRP may not clearly reflect the progression of internal angiogenesis and inflammation, at least in a subset of patients. These observations suggest that different factors might be responsible for inducing progression of inflammation in early RA patient which generally may not be well evaluated only by DAS28 CRP scores.

The appearance of increased PDUS vascularity has been well corroborated with an enhanced level of VEGF and TNF-*α* in both moderate and high DAS28 patients, indicating an early induction of angiogenesis. Few studies were performed earlier to find the correlation of serum angiogenic markers with PDUS detected synovitis in patients with inflammatory arthritis. Świdrowska J. et al. assessed the serum or synovial fluid levels of angiogenic markers in early Juvenile idiopathic arthritis (JIA) patients to find their relevance to disease activity or degree of ultrasound detected synovial inflammation and angiogenesis. They found a significant correlation of joint vascularization intensity with higher serum VEGF. However, oligoarticular JIA was the major subtype of the study population (69.8%) and remaining was polyarticular JIA [22]. In another important study with 12 DMARD naïve early RA patients, Kelly S. et al. analyzed the relationship of US detected synovitis and synovial vascularity with various angiogenic, lymphangiogenic factors and cellular inflammatory mediators. Apart from clinical, US, and biochemical assessments, US guided biopsy from suprapatellar pouch was done. They showed a good correlation of PDUS with VEGF-A, Angiopoietin-2, and Tie-2 [23]. In this study wrist joint ultrasound was not done. We conducted PDUS of wrist joints as this is very commonly involved in RA and is mostly studied by PDUS in patients with RA. However, these studies were not primarily meant for comparison between PDUS vascularity and DAS28 CRP with respect to their association with inflammatory and angiogenic mediators.

PDUS has gained its acceptance as an important tool for detection of early synovial inflammation and also to monitor treatment response. DAS28 is a very useful composite measure of RA disease activity index which is commonly used in routine practice to stratify patients within different disease activity states and also to monitor treatment response. This study was the first of its kind to compare these two grading systems with respect to the expression of angiogenic and inflammatory mediators in early RA. Discordance of these two scoring systems (grade 2 and grade 3) has been documented in literatures but most of these studies are concerned with treated RA patients who are in a state of clinical remission. Ramírez J. et al. found ultrasound-defined synovitis in almost half of the RA patients in clinical remission state as per DAS-28 ESR score. Importantly patients with USG defined synovitis also expressed higher serum levels of the angiogenic biomarkers [24]. This finding was further reinforced by the results of the APPRAISE study in which PDUS findings did not show correlation with DAS-28 CRP derived response in RA patients treated with Abatacept [11]. However, there is paucity of data regarding this issue in DMARD naïve early RA patients. In our study, logistic regression analyses revealed that angiogenic and inflammatory mediators were actually represented better with PDUS vascularity grade than DAS-28 CRP in DMARD naïve early RA patients. Ang-1, Ang-2, and VEGF levels were found to be higher in moderate DAS-28 CRP patients with grade 3 PDUS vascularity even than those with high DAS-28 CRP but grade 2 PDUS score. TNF-*α* was relatively high in both moderate and high DAS28 patients indicating an early induction of this cytokine at the onset of disease progression.

Results of this study depicted that only DAS-28 CRP analysis might not be robust enough to represent the actual underlying inflammatory burden, at least in some subgroup of early RA population. Application of PDUS vascularity grading may be more informative in this scenario to identify patients with higher level of inflammatory markers. This may be also helpful from the therapeutic view point.

To the best of our knowledge, no comparative analyses have been performed to assess the relationship between angiogenic and inflammatory mediators with PDUS vascularity grading and DAS28-CRP in early RA patients.

## Figures and Tables

**Table 1:** Baseline characteristic of the RA patients and healthy controls.

**Variables**	**RA patients (*n=50*)**	**Healthy controls (*n=30*)**	***P *value**
Age(Years)	35.90±18.607	34.03±10.3	0.7
Gender(M/F)	4/46	2/28	0.8
Disease duration (Months)	4.280±2.821	-	-
BMI(kg/m2)	36.15±9.400	34.90±13.51	0.4
Anti CCP(RU/ml)	215±164.1	_	_
**CRP(>0.3mg/l)**	21.14±15.89	0.23±0.14	**<0.0001**
VAS(0-100)	58.54±19.78	_	_
DAS 28-CRP	5.175(4.303-5.715)	_	_
HAQ-DI	2.559±0.94	_	_

Clinical and serological data of 50 early RA patients with synovitis and 30 individuals of healthy control. Data expressed as mean ± SD, median (interquartile range). BMI = basal metabolic rate. Anti-CCP = anti-citrullinated protein antibody; CRP = C-reactive protein; VAS = Visual Analogue Score; DAS 28-CRP = disease activity score 28 CRP; HAQ-DI = health assessment questionnaire disability index; *p*< 0.05 is considered significant.

**Table 2:** Expression of angiogenic and inflammatory markers in RA patients and controls.

**Parameters(pg/ml)**	**RA patients (*n=50*)**	**Healthy controls*(n=30)***	***P* value**
ANG-1	6501±5343	224.0±126.0	<0.0001
ANG-2	2292±2234	220.686±123.2	<0.0050
TNF-*α*	823.2±613.5	37.63±35.36	<0.0001
IL-6	147.3±129.6	7.686±7.136	<0.0001
VEGF	200.3±153.5	26.10±22.10	<0.0001

Laboratory findings of vascularity and inflammatory markers of 50 early RA patients and 30 individuals' healthy controls. Data expressed as mean±SD. ANG-1= Angiopoietin-1, TNF-*α* = tumor necrosis factor, IL-6= interleukin-6, and VEGF= vascular endothelial growth factor.

**Table:** (a) Logistic regression analysis of PDUS with selected clinical features

**Dependent Variables**		**PDUS**				
** R** ^**2**^		**0.5320**				
**Significance Level**		**0.0001**				
**Variable**	**Coefficient**	**Std.Error**	**Wald**	***P***	**Odds ratio**	**95**%**CI**
ANG-1	-0.0066069	0.0038852	2.8919	0.0890	0.9934	0.9859 to 1.0010
ANG-2	-0.000063268	0.000086613	0.5336	0.4651	0.9999	0.9998 to 1.0001
TNF-*α*	0.0012117	0.00055438	4.7775	**0.0288**	1.0012	1.0001 to 1.0023
VEGF	0.0079439	0.0035127	5.1142	**0.0237**	1.0080	1.0011 to 1.0149

**Table:** (b) Multiple linear regression analysis of DAS28 CRP with selected clinical features

**Dependent Variables**		**DAS28 CRP**			
**Coefficient of determination R** ^**2**^		**0.1**			
**R** ^**2**^ **-adjusted**		**0.02**			
**Multiple correlation coefficient**		**0.32**			
**Model 1**					
**Independent variables**	**b**	**Std Error**	^**r**^ **partial**	**t**	***p* value**
ANG-2	0.0003466	0.0009471	0.05572	0.366	0.7162
ANG-1	0.00002395	0.00002459	0.1469	0.974	0.3355
TNF-*α*	0.0001029	0.00008316	0.1854	1.237	0.2228
VEGF	-0.0005151	0.0003407	-0.2247	-1.512	0.1378

*R* ^2^, multiple coefficient of determination; b standard regression coefficient. For abbreviations refer to Table 2.

## Data Availability

The data used to support the findings of this study are available from the corresponding author upon request.
